# Low-dose trofosfamide plus rituximab is an effective and safe treatment for diffuse large B-cell lymphoma of the elderly: a single center experience

**DOI:** 10.1186/s12885-018-4885-5

**Published:** 2018-10-19

**Authors:** Roland Christian Schelker, Wolfgang Herr, Albrecht Reichle, Martin Vogelhuber

**Affiliations:** Department of Internal Medicine III, Hematology & Oncology, University Hospital Regensburg, Franz-Josef-Strauss-Allee 11, 93053 Regensburg, Germany

**Keywords:** Diffuse large B-cell lymphoma, Rituximab, Trofosfamide, Elderly patients

## Abstract

**Background:**

Rituximab plus combination chemotherapy with cyclophosphamide, doxorubicin, vincristine, and prednisone (R-CHOP) is broadly accepted as standard for the treatment of diffuse large B-cell lymphoma (DLBCL). Nevertheless, there is sparsely data concerning the management of elderly patients.

**Methods:**

We performed a retrospective study of treatment with rituximab and low-dose trofosfamide in elderly patients (≥ 75 years) with DLBCL who were not suitable for R-CHOP or R-CHOP-like regimens or who did not consent to aggressive treatment. The choice regarding the qualification for R-CHOP or R-CHOP-like regimen was left to the estimation of the treating physicians.

**Results:**

Eleven patients with a median age of 83 years (range, 75–90 years) were included. The age-adjusted international prognostic index was low risk in one patient, low-intermediate in four patients, high-intermediate in three patients, and high risk in 3 patients. All patients were evaluable for response. Five patients (45%) achieved a complete response, three (27%) a partial response, one (9%) stable disease, and two (18%) progressive disease. The estimated 1-yr overall survival was 54.5%, and the estimated 1-yr progression-free survival 45.5%, however, three patients (27%) were alive without evidence of disease at 16–20 months from start of treatment. Main toxicity was leukopenia (36% grade III or IV), whereas grade III/IV non-hematological adverse events did not occur.

**Conclusions:**

Due to its potency and low toxicity, trofosfamide/rituximab might represent an alternative therapy for DLBCL of elderly patients not suitable for R-CHOP. This observation, however, should be confirmed in a larger patient population within a prospective clinical trial.

## Background

Diffuse large B-cell lymphoma (DLBCL) is by far the most common subtype of high-grade B-cell non-Hodgkin lymphoma (B-NHL) in adults. Worldwide, the current annual incidence of DLBCL is estimated to be 7 cases per 100,000 persons [1]. This incidence continually increases with age, and approximately 40% of cases occur in patients elder than 70 years [2]. Due to the global ascent of the older population, an additional rise of the absolute count of DLBCL cases can be anticipated, particularly in very old people. Retrospective data have already delineated that the prognosis of elderly patients is worse than that of younger patients, the choice of adequate treatment modalities being a real challenge [3–6].

It is not clear if DLBCL in the elderly carries a different genotype than in younger patients but it was demonstrated that molecular features with distinct prognosis are associated with age [7]. Nevertheless, DLBCL in elderly patients is not considerably less responsive to treatment than in younger patients, and the principal reason for the poor prognosis of very old patients is their diminished ability to tolerate treatment [8]. The number of treatment-related adverse events is heightened by limited bone-marrow function, altered drug metabolism, and existence of comorbid diseases. Various efforts to reduce doses of the standard chemotherapy regimen CHOP (cyclophosphamide, doxorubicin, vincristine, and prednisone) or to replace the components with less toxic drugs have diminished toxicity but did not ameliorate survival [9–11].

The major therapeutic advancement made by incorporation of the monoclonal antibody rituximab with CHOP (R-CHOP) has modified the treatment outcome of patients with DLBCL dramatically. A randomized open-label trial by the Groupe d’Etudes des Lymphomes de l’Adulte (GELA) in patients aged 60–80 years showed significantly superior complete response and survival rates in patients treated with CHOP and Rituximab compared to CHOP alone, without a clinically increase in toxicity [12–14]. These results were reproduced by two randomized trials in a similar patient population [15, 16].

Since then, incorporation of rituximab in R-CHOP-like regimens has been examined for more frail elderly patients in several phase II trials [17–26], demonstrating efficacy and tolerability. Nevertheless, the rate of circumstances incapacitating patients from R-CHOP-like regimens is elevated in elderly population. Hence, alternative efficient treatment options with less toxicity are needed. Such treatments, accompanied by supportive measures and frequent toxicity monitoring, can be more than palliative and add considerable quality/quantity of life.

In the pre-rituximab era, the findings of phase II studies with metronomic low-dose trofosfamide (50 to150 mg/day) in patients with relapsed or refractory NHL, who did have a palliative treatment option, were published, but these studies revealed only an overall response rate (ORR) of 50% and median response duration of 4 months in a small cohort of NHL patients, including DLBCL [27–30]. Few long-term complete remissions (7–10 months) were observed, even in patients with refractory disease [29]. Toxicity was generally mild despite bone marrow depression with leukopenia up to grade III. In one particular case of relapsing anaplastic large-cell lymphoma trofosfamide treatment resulted in ongoing complete remission 16 months after withdrawal of the drug [31]. Since these findings proved potency of trofosfamide in NHL and as the drug was very well-tolerated yet in older patients [32], compassionate use of low-dose trofosfamide and rituximab (R-T) in elderly patients who were not eligible for standard immunochemotherapy, was conducted. Notably, rituximab is approved for first-line treatment of CD20^+^ NHL worldwide, while trofosfamide is approved in Germany for treatment of NHL after failure of standard chemotherapy.

Trofosfamide is an alkylating agent of the group of oxazaphosphorines [33]. A number of preclinical and clinical findings showed that pharamacodynamics and the mode of action of the medication considerable vary from classical alkylating agents like cyclophosphamide, which is part of the CHOP regimen. Contrary to the other oxazaphosphorines, trofosfamide is more lipophilic and is only disposable as an oral formulation [34]. In vitro examination revealed crosslinks between DNA and chromosomal proteins and showed some antiangiogenetic potential in the metronomic schedule [35, 36]. Trofosfamide is a prodrug, known to be metabolized mainly to ifosfamide and merely to a small rate to cyclophosphamide [37]. Recently, Brinker et al. revealed a third pathway in the metabolism of trofosfamide to 4-hydroxy-trofosfamide, which seems to be the most important one [34].

Trofosfamide has evinced a wide efficacy in the therapy of multitudinous hematological and solid tumors [38]. Currently, there is no randomized trial to compare trofosfamide with standard therapy. Moreover, thus far no data exist, associating rituximab to trofosfamide treatment.

Therefore, we here present the first study that investigates trofosfamide in combination with rituximab for the treatment of patients with DLBCL and also the first report on trofosfamide given as first-line therapy in this disease entity.

## Methods

### Patients

In this study, we conducted a retrospective investigation of 11 cases diagnosed with DLBCL who were treated in first-line (*n* = 4), second-line (*n* = 6) and third-line (1) therapy with R-T chemoimmunotherapy at University Hospital Regensburg. Medical files of cases trailed between March 2014 and June 2017 were examined. Informed consent was obtained from each patient that drugs, approved for lymphoma therapy should be used, which have not been tested in combination, yet.

In every patient, histological evaluation and appropriate immunophenotyping assays were conducted on tissue specimen recieved from lymph node extirpations and resections of extranodal manifestation sites including endoscopic biopsies and bone marrow (BM) biopsies, and diagnosis was made conformable to the World Health Organization (WHO) classification for malignancies of lymphoid origin [39].

The following clinical and histological information was compiled: age, symptoms, performance status (PS), clinical stage, extent of extranodal, nodal and BM involvement, lactate dehydrogenase (LDH), International Prognostic Index (IPI), proliferation index, prior treatment, status before rituximab/trofosfamide, and treatment duration in months. The IPI score was documented agreeable to the established system [40]. The staging was performed according to the Ann Arbor staging criteria [41].

In all cases, appropriate clinical assessment before R-T immunochemotherapy, during therapy in intervals of 3 weeks (one cycle), at progression, withdrawal of consent, severe side effects, every month after achieving CR and during post-treatment follow-up (every 3 months) was performed. Before starting therapy, the evaluation comprised at least a physical investigation, hematological and chemical laboratory examination and positron emission tomography-computed tomography (PET-CT) [or contrast-enhanced computed tomography (CT) of chest, abdomen and pelvis]. Supplementary BM biopsy was done in cases with BM infiltration to evaluate the efficacy of the therapy. In follow-up clinical and laboratory assessment was performed before start of the next cycle. Response was assessed after every 2 cycles (3 weeks/cycle) of therapy and after stop of therapy of any cause in line with the 2007 International Working Group revised response criteria [42]. Patients were categorized conformable to most favorable regress: complete response (CR), partial response (PR), stable disease (SD), or disease progression (PD). The ORR was defined as the population who acquired a CR or PR, respectively. Toxicities were recorded and were ranking according to the Common Toxicity Criteria for Adverse Events v3.0 [43].

The R-T treatment comprised rituximab (MabThera®, Roche Germany) administered at 375 mg/m^2^ intravenously (at first administration) and 1400 mg subcutaneously (subsequent administrations) on day 1 of every cycle and trofosfamide (Ixoten®, Baxter Oncology Germany) given at a metronomic (low) dose of 50 mg (1 patient), 100 mg (8 patients; two single doses of 50 mg) or 150 mg (two patients; three single doses of 50 mg) daily by oral administration on day 1–21. In the two patients who started with trofosfamide 150 mg daily, dose adaption to 100 mg daily was necessary due to cytopenia during the course of treatment. Prior to the application of rituximab, premedication with 4 mg dimetindene was administered intravenously. Rituximab was given in 21-day cycles up to 8 cycles, trofosfamide until PD, cytopenia or impaired PS and 10 cycles after achieving CR. Rituximab was given in the out-patient clinic. The median treatment duration was 5 months. Granulocyte-colony-stimulating factor (G-CSF) was not administered.

The retrospective patient analysis was performed in accordance with the current version of the Helsinki Declaration and the use of oral consent had been approved by the local Ethics Committee of Human Experimentation, University Regensburg, documented by the notification with the reference number: 17–667-104. All cases were anonymized and their medical files were investigated anonymously. Informed consent for publication of anonymized medical files for research purpose was received orally from every patient.

### Statistical analysis

Analyses were performed using PRISM 7 (Graphpad, San Diego, CA, US) statistical software. Patient features are showed as descriptive statistics, with numbers for categorical variables. Kaplan-Meier survival evaluation was conducted appreciate the progression–free survival (PFS) and overall survival (OS). PFS was monitorized from starting R-T treatment to PD/relapse or death. Length of follow-up was calculated from the moment of beginning R-T treatment to the final follow-up or time of death.

## Results

### Patient characteristics

We conducted an electronically quest in the catalogue of the University Hospital Regensburg. Altogether 11 cases with histologically-evidenced DLBCL treated with R-T from February 2014 to June 2016 were perceived. Patients were registered successively with the intention to prevent selection bias.

Table 1 recapitulates the patient features at diagnosis, prior treatment regimen and the status at first application of R-T. The median age at treatment onset was 83 (range 75 to 90) years. At the beginning of the investigation, 55% had a favorable (ECOG 0–1) and 45% a compromised (ECOG 2–3) PS. LDH was elevated in 27% of the patients. Conformable to Ann Arbor criteria, 18% were staged as stage I, 18% as stage II, 18% as stage III and 45% as stage IV. In all but three of the patients extranodal manifestations and associated clinical symptoms could be identified. Thereof, 63% presented with extra-lymphatic manifestations including the left thoracic wall, the gluteal muscles, the left kidney and the left adrenal gland, both mammary glands, the spleen, the liver, the bones inclusively the BM, cerebral and meningeal dissemination and cutaneous infiltration. According to IPI, 9% were at low risk, 36% were at low-intermediate risk, 27% at high-intermediate risk and 27% at high risk. Expression of CD20 in lymphoma tissue could be detected in all patients by immunohistochemical analyses, while only 9% expressed CD5, 55% CD10, 9% CD30, 18% CD79a, 9% CD138, 82% BCL2, 55% BCL6 and 36% MUM1. Median proliferation index Ki-67 or MiB1was 83%, ranging from 40 to 95%.

**Table 1:** Patients characteristics

ID	stage	IPI risk	PS	extranodal involvement	immunohistochemical expression	LDH at diagnosis in U/l (Normal < 250 U/l)
1	II	low	*1*	oropharynx, base of the tongue	CD5, CD10, CD20, BCL2	122
2	III	high	*1*	N	CD10, CD20, CD30, BCL2, BCL6	369
3	II	high-intermediate	3	N	CD20, BCL2, BCL6, MUM1	718
4	I	low-intermediate	*2*	stomach	CD10, CD20, BCL6	184
5	IV	high	1	left thoracic wall, gluteal muscles, left kidney, spleen, liver	CD10, CD20, BCL2, BCL6, MUM1	312
6	III	high-intermediate	*2*	bone marrow	CD20, CD79a, BCL2, BCL6, MUM1	180
7	IV	high-intermediate	2	os ileum, left sacroiliac joint, lumbar vertrebal body 5, brain	CD10, CD20, CD79a, CD138, BCL2	196
8	IV	high	2	skin, meninges, bone marrow	CD20, BCL2	164
9	I	low-intermediate	0	both mammary glands, left adrenal gland	CD20, CD79a, BCL2, MUM1	150
10	IV	low-intermediate	*1*	N	CD20, BCL2	205
11	I	low-intermediate	1	right tonsill	CD10, CD20, BCL6	209

*IPI* International Prognostic Index, *PS* performance status, *N* negative, *CD* cluster of differentiation, *BCL* B-cell lymphoma, *MUM* multiple myeloma oncogene, *LDH* lactate dehydrogenase

### Treatment

All 11 patients obtained R-T therapy as scheduled. In consensus with our institutional guideline the indication for this immunochemotherapy regimen was as follows: First-line treatment was indicated in patients being not eligible for R-CHOP or R-CHOP-like regimens due to multiple comorbidities, poor PS or higher age; rescue treatment with R-T was indicated in patients with PD or relapse after R-CHOP or R-CHOP-like regimens. Some patients (27%) were extensively pretreated: 5 cycles of B-ALL/NHL regimen of the German ALL study group [44] (rituximab, cyclophosphamide, methotrexate, vindesine, etoposide, doxorubicin, cytarabine); 6 cycles of R-mini-CHOP [21] + 6 cycles R-TPIP (rituximab, trofosfamide, procarbazine, idarubicin, prednisone); 6 cycles R-CHOP + 14 × rituximab maintenance. Moreover, in single patients pre-treatment with cyclophosphamide (*n* = 2) or one cycle R-HOP (*n* = 1) was performed prior to R-T due to bulky disease.

### Response and follow-up

The response data is specified in Table 2. The ORR was 73% with 5 CR and 3 PR. One patient developed SD and the remaining two patients had PD. Among the patients who achieved CR, 40% presented complete disappearance of lymphoma after 6 cycles, whereas the other 60% developed CR after 8 cycles of R-T therapy. It is noteworthy that in 45% of the patients the incipient symptoms completely disappeared, while 36% showed a reduction of symptoms and the patients who presented PD did not improve at all.

**Table 2:** Response and side effects

ID	response	grade III/IV side effects	
		hematologic	non-hematologic
1	PR	no	no
2	PR	pancytopenia	no
3	CR	leukopenia	no
4	CR	no	no
5	CR	pancytopenia	no
6	CR	no	no
7	SD	no	no
8	CR	leukopenia	no
9	PD	no	no
10	PR	no	no
11	PD	no	no

*PR* partial response, *CR* complete response, *SD* stable disease, *PD* progressive disease

**Fig. 1:**
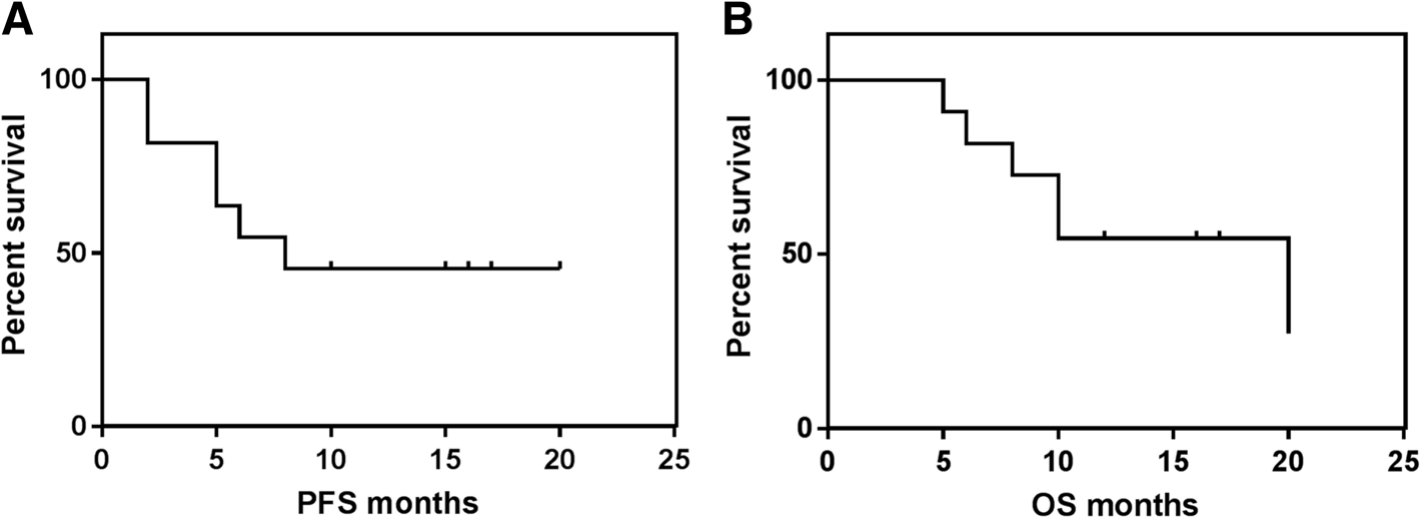
**a** Kaplan-Meier curves on progression-free survival (PFS) and (**b**) overall survival (OS) for patients (*n* = 11) who were treated with the R-T regimen

**Fig. 2:**
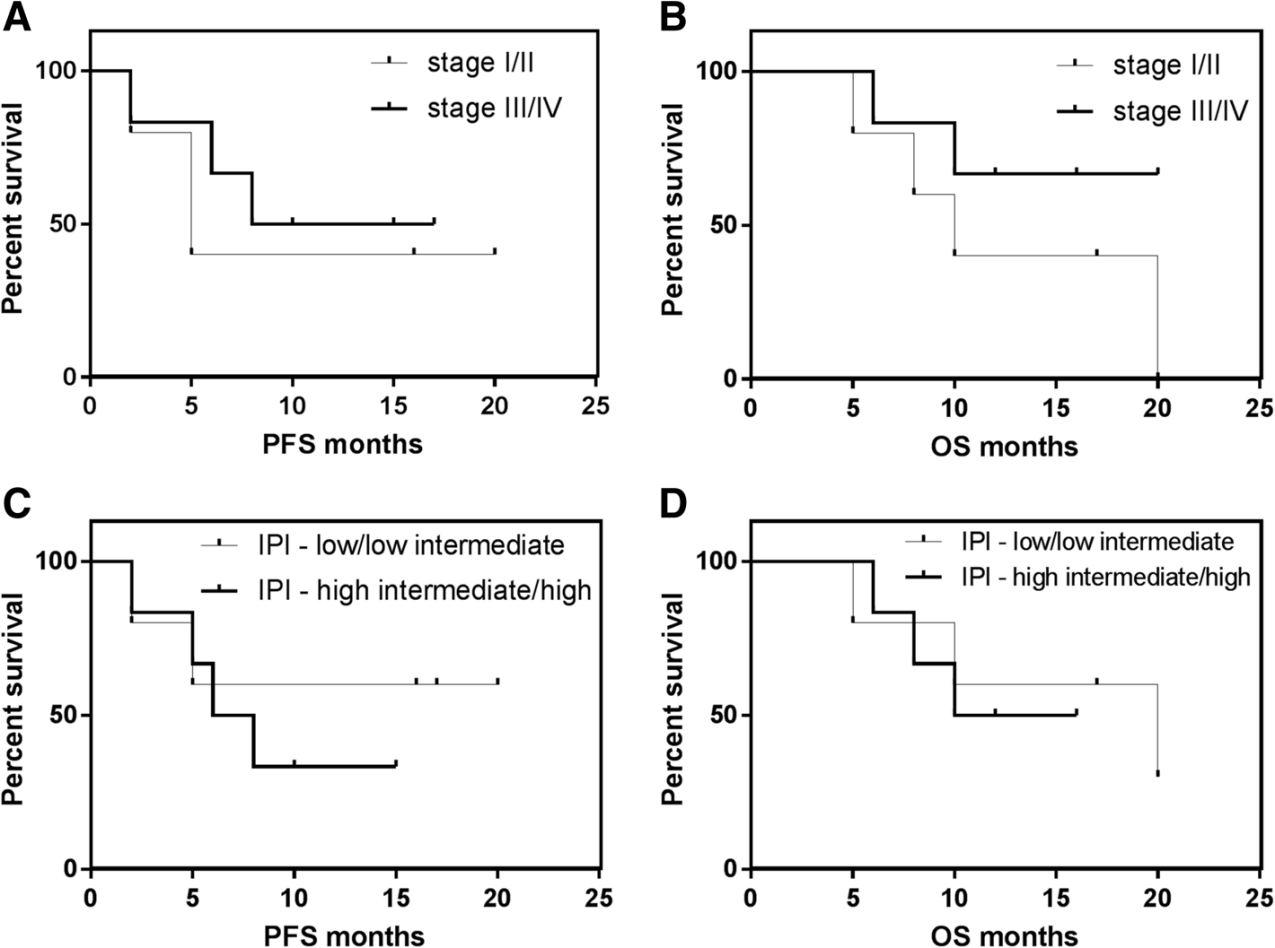
Kaplan-Meier curves on progression-free survival (PFS) and overall survival (OS) with regard to Ann Arbor staging (**a**, **b**) and age-adjusted international prognostic index (**c**, **d**)

Of the 5 patients who achieved CR, 60% could maintain this status even long time after cessation of treatment (7–14 months after discontinuation of R-T), the remaining 40% endured cerebral PD 1–3 month after attaining CR. Of the patients who developed PR 67% progressed with extranodal PD (enoral, ileum) after 3–8 months of continuous R-T treatment, 33% remained in stable PR for 6 months. One patient had SD without cessation of therapy. Two cases had PD at the first staging after 2 months on R-T. At a median observation time of 12 months, 73% of the patients were alive, and 27% were in sustained CR. The estimated PFS at 1 year was 45.5% with an estimated median duration of 9.4 months (Fig. 1a). The estimated 1-yr overall survival (OS) was 54.5% (Fig. 1b). Six patients survived longer than 12 months, with only one of them dying 20 months after beginning R-T therapy due to PD. A subgroup analysis of our data (Fig. 2a, b, c and d*)* suggested that a benefit from treatment with R-T not alone occurred in cases with limited stages or low IPI, but also in three patients with stage IV and two patients with high-risk/one with intermediate-high risk corresponding to IPI who were alive in CR (*n* = 2) or SD (*n* = 1) 16 months or longer after treatment was started.

### Toxicity

Median time on therapy was 5 months. Therapy-associated toxicities were smooth and endurable (Table 2). Hematologic toxicities were basically noticed, with leukopenia grade III and IV in 36% of the patients. After interruption of therapy, hematology was followed weekly, and treatment with trofosfamide restarted at one dose level lower (reduction of 50 mg daily) when leukopenia had resolved to ≤ Grade II. In all cases leukopenia resolved within 3 weeks. Grade I nausea and dyspnea was observed in 9% during administration of rituximab, grade I fatigue and anorexia in other 9% during R-T therapy. One patient reported cramps in the leg. No incident of febrile neutropenia resulting in hospitalization occurred.

## Discussion

Our retrospective clinical study suggests that the R-T regimen is effective and well-tolerated in DLBCL of elderly and comorbid patients, and additionally, as three of the cases are still alive in the absence of lymphoma 16, 17 and 20 months upon treatment, respectively, they suggest that the treatment could be even curative in a fraction of patients with ongoing CR. The survey was not planned to substitute the latest standard, R-CHOP, in older patients [45], nor should it replace R-CHOP-like regimens: nonpegylated liposomal doxorubicin instead of standard doxorubicin (R-COMP) [17, 18]; combination of rituximab and nonpegylated liposomal doxorubicin (R-NPLD) [19]; replacement of conventional doxorubicin by pegylated liposomal doxorubicin [20]; R-mini-CHOP [21]; a dose-adjusted infusional regime (DA-POCH-R) [22]; induction R-CNOP or R-CVP for three cycles, followed by maintenance rituximab in responders [23]; substitution of gemcitabine for anthracycline in a R-CHOP-like regimen (R-GCVB) [24]; combination of ofatumumab and reduced-dose CHOP [25]; induction R-EPOCH or R-CEOP [26]. However, the primary motivation for this retrospective study has been to define an adequate therapy for patients, who were classified by their hematologists not to be suitable for repetitive R-CHOP or R-CHOP-like regimens. Actually, Thieblemont et al. [5] highlighted in their study of NHL cases elder than 80 years that a treatment choice was frequently difficult. In their study of 205 cases, merely 4% had obtained standard treatment with CHOP or R-CHOP. This evaluation shows that in the global population of older patients with highly malignant lymphomas, a significant number is not categorized by their doctors to be eligible for CHOP or R-CHOP and hence, other potentially less toxic combinations are necessary. Nevertheless, for this population, there is a lack of data disposable to direct treatment decision.

The CR proportion of 45% in the current retrospective data analysis appears inferior than in a group of cases elder than 75 years who received R-CHOP; nevertheless, one must take into consideration that a patient cohort like this follows intense screening and solely patients with a superb PS and few comorbidities will finally undergo therapy with R-CHOP. Regarding R-CHOP-like regimens, the CR rate appears comparable or even better (R-CVP: 37%; R-GCVB 39%) [24, 46], and the toxicity profile seems more moderate for R-T. Thus R-T combines both, efficacy and tolerability in comorbid elderly DLBCL patients.

In a prospective phase II study by Park et al. applying a regimen based on bendamustine and rituximab (B-R), a group of 23 patients with a median age of 80 years was examined for efficacy and toxicity. The response rate was similar to our work, with 78% ORR and 52% CR. In general, the treatment was excellent tolerated; though, sixteen patients deceased in the course of the study period, four of them treatment-related [47]. These results are comparable to recently published retrospective or prospective studies of B-R in the elderly [48, 49]. To our knowledge, B-R is the only published regimen alternative to R-CHOP or R-CHOP-like regimens excepting the findings reported in our study in elderly cases with DLBCL and this scarceness of information emphasizes the necessity for subsequent studies in this age-cohort. Experience with distinct combination therapies and integration of geriatric scores are required to ease the adjudication, in whom a standard procedure could be beneficial and in whom other options could be the more favorable selection.

A subgroup examination of our data suggests that high-risk patients may also benefit from treatment with R-T. However, based on the small number of patients studied, it was not feasible to pick out any factor that would predict cases with beneficial outcome after therapy with R-T. In the study by Thieblemont et al. [5], IPI and Ann Arbor staging were separate prognostic factors in a multivariate analysis. This is presumably also the case for patients treated with R-T; however, our report suggests that R-T could be effective even in a significant population with advanced-stage and/or unfavorable IPI. We would like to emphasize, that R-T was effective with trofosfamide even at low dose of 50–100 mg daily.

Taking into account the seniority of our cohort, the toxicity of R-T was quite mild. Compared to the studies with the B-R regimen in DLBCL of the elderly [47, 48], grade III and IV non-hematological adverse events could not be detected. Adverse events were principally hematologic toxicities, with grade III and IV leukopenia in 4/11 (36%) patients. In comparison with studies utilizing trofosfamide monotherapy [27–30] in patients with DLBCL, hematologic and non-hematologic toxicity were greatly alike, which confirmed the relatively mild toxicity of this regimen. Notably, the low rate of hematotoxicity in the present report was achieved, although G-CSF was not administered. Furthermore, the R-T combination does not lead to alopecia. Patients with a poor PS were, contrary to the study on the B-R regimen by Weidmann et al. [48], not excluded in our work; these patients could be precisely the subgroup that most probable benefits from R-T since cardiac toxicity is very low, and additionally, the combination is excellent tolerated by older patients as shown in this report.

## Conclusions

In summary, R-T is an effective and well-tolerated treatment regimen and hence should be considered particularly in older and/or comorbid patients with DLBCL who are not suitable candidates for R-CHOP or R-CHOP-like regimens. Nevertheless, the number of patients in this study is low, and therefore, our findings have to be validated in larger study populations. Moreover, the retrospective nature of the study makes the adverse events difficult to interpret and difficult to compare to other regimens. Therefore, future trials should be designed prospectively and comprise geriatric assessment scores which could assist to recognize subgroups of DLBCL patients who will benefit from treatment with R-T.

## Abbreviations

BM: bone marrow
B-NHL: B-cell non-Hodgkin lymphoma
B-R: bendamustine and rituximab
CHOP: cyclophosphamide, doxorubicin, vincristine, prednisone
CR: complete response
CT: computed tomography
DLBCL: diffuse large B-cell lymphoma
G-CSF: granulocyte-colony-stimulating factor
GELA: Groupe d’Etudes des Lymphomes de l’Adulte
IPI: International Prognostic Index
LDH: lactate dehydrogenase
ORR: overall response rate
OS: overall survival
PD: disease progression
PET-CT: positron emission tomography-computed tomography
PFS: progression-free survival
PR: partial response
PS: performance status
R-CHOP: rituximab with CHOP
R-NPLD: rituximab with nonpegylated liposomal doxorubicin
R-T: rituximab and trofosfamide
R-TPIP: rituximab, trofosfamide, procarbazine, idarubicin, prednisone
SD: stable disease
WHO: World Health Organization
